# Learning Difficulties and Loneliness in College and Beyond: The Mediating Role of Self-Efficacy, Proactive Coping, and Hope

**DOI:** 10.3390/ijerph181910508

**Published:** 2021-10-07

**Authors:** Tamar Icekson, Oranit Davidson Begerano, Michal Levinson, Jenny Savariego, Malka Margalit

**Affiliations:** 1 School of Behavioral Sciences, Peres Academic Center, 10 Peres St., Rehovot 7610202, Israel; icekson@post.bgu.ac.il (T.I.); odavidson@pac.ac.il (O.D.B.); michal.sapv@gmail.com (M.L.); jennysav@gmail.com (J.S.); 2 Department of Management, Ben-Gurion University, Beer Sheva 8410501, Israel; 3 School of Education, Tel-Aviv University, Tel-Aviv 6997801, Israel

**Keywords:** learning difficulties, loneliness, self-efficacy, proactive coping, hope

## Abstract

Following the conservation of resources, social-cognitive and hope theories, the goals of this study were to identify the role of self-efficacy, proactive coping, and hope as mediators in the relations between learning difficulties and loneliness distress. A questionnaire was sent to current and past students. The sample consisted of 498 participants. The results demonstrated that individuals with learning difficulties reported higher levels of loneliness compared with individuals without learning difficulties. Moreover, self-efficacy, proactive coping, and hope mediated the relations between levels of learning difficulties and loneliness. Specifically, the final model emphasized the important role of hopeful beliefs, since hope mediated the relations between learning difficulties, self-efficacy, and proactive coping with loneliness. In terms of practical implications, professionals’ awareness as well as psychoeducational programs could be tailor-made to enhance hopeful beliefs and reduce loneliness.

## 1. Introduction

Learning disorders do not end with school years. Many struggles such as difficulties to pay attention for an extended time, difficulties to remain seated, slow reading and memory difficulties may continue to be a challenge in colleges and working environments, and be a source of social and emotional distress [1,2]. Some individuals develop strategies to cope successfully with the new challenges in postsecondary education and the workplace. Many others continue to feel distressed and experience self-doubt regarding their opportunities for success in those new environments [3]. This prolonged distress, combined with lingering memories of academic struggles often predict lower self-efficacy and higher levels of loneliness [4]. Thus, it is not surprising that loneliness affects more often people with varied learning difficulties and disabilities [5].

Previous studies have reported the risk of loneliness among children and adolescents with learning disorders [6], documenting their challenging experiences in facing academic and social encounters [7,8]. Similarly, individuals with chronic academic difficulties often report higher levels of loneliness [9]. There is a need to identify mediating mechanisms that may explain this relationship during their college studies and afterwards. Thus, the current study focuses on adults who experience consistent learning difficulties due to various causes such as learning disorders and ADHD during and after their school years, in order to examine their relations with loneliness. Since loneliness is considered a stressful experience [10], personal resources may provide strength and emotional inoculation that may lead to a better and effective coping with stress and a reduced loneliness distress. Hobfoll’s [11] conservation of resources (COR) paradigm offers a conceptual model for understanding the mediating mechanisms of resources to reduce suffering. Research already documented the relations between personality characteristics and loneliness [12] focusing attention on the need to explore how individual differences and loneliness are interwoven

According to the COR theory [11], a significant and ongoing drain on resources results from a prolonged need to cope with difficulties and chronic stressors. Resources are defined as “entities that either are centrally valued in their own right, or act as means to obtain centrally valued ends” ([11], p. 307). COR theory posits that individuals strive to increase their resources, and that “resources tend to generate and activate additional resources, thus creating ‘resource caravans’, which may result in positive outcomes and well-being” ([11], p. 312). Thus, people who possess greater personal resources can better recover from losses and are more capable of acquiring additional resources to obtain further goals [13].

In line with COR theory, the goals of this study are to identify the protective role of the future oriented personal strengths (self-efficacy, proactive coping, and hope) as factors that may mediate the relations between experiencing learning difficulties and loneliness distress in college (e.g., [1,13] and beyond [14]).

### 1.1. Loneliness

Loneliness has been conceptualized as a distressful experience reflecting unmet personal and interpersonal needs. It has been related to unsatisfied social relations, and associated with social alienation, depression, and poor social skills [15]. It has been related to individual characteristics, personal difficulties, and social belonging and interpersonal connections [16]. Research also demonstrates the relations of the loneliness experience to a wide range of sociodemographic aspects [17].

Strong evidence shows that social connections have been considered significant protective factors, and loneliness as a risk factor for health and wellbeing [18]. According to Cigna’s 2020 Loneliness Index, the proportions of this source of distress are growing with significant outcomes to wellbeing [18,19]. Lonely workers reported being twice as likely to miss a day of work due to illness, and five times as likely to miss a day of work due to stress. Lower productivity and lower quality of work were testified, as well as a higher risk of staff turnover. Loneliness may result from struggling with learning challenges and feeling different and less accepted by peers. Indeed, previous studies documented that adolescents as well as young adults with learning disabilities reported higher levels of loneliness [20,21].

A comprehensive review of studies conveyed varied individual differences that characterized lonely people, such as maladaptive cognitive biases, low self-worth, and various personality traits. Most of them focus attention on the past experiences [22]. Thus, several resources may be considered protective resources, such as resilience. Yet, in our theoretical model, we expected that future oriented protective factors may promote a dynamic change in the distressful loneliness. Thus, in the current study three future orienting empowering strengths were selected as mediators for reducing the risk of loneliness: self-efficacy, proactive coping and hope. In line with COR theory [11] we expect that higher levels of self-efficacy will promote proactive coping and both of them will enhance hope. Together they may facilitate the resource gain, resulting in a reduced loneliness. The role of these protective factors in explaining the relationship between prevalent disabilities and the experience of loneliness among individuals has not been fully explored.

### 1.2. Self-Efficacy

Self-efficacy refers to the individuals’ belief and confidence in their ability to execute behaviors necessary to produce successful performance [23]. Perceived self-efficacy refers to the confidence of being able to achieve desired outcomes. People’s beliefs in their efficacy affect the type and level of goals that they set for themselves and the strength of their commitment to achieve them [24]. Individuals with higher perceived self-efficacy were found to better perform in college and after graduation [25]. Some students with learning disorders were found to be able to gradually develop their abilities to cope with their difficulties and to experience successes that would further increase their efficacy beliefs regarding their future achievements in different environments. Many others may continue to experience self-doubt regarding their abilities, learning skills, and future opportunities for success, predicting lower self-efficacy [1,26]. Hobfoll [12] suggested that those who possessed high levels of self-efficacy perceived themselves as more capable of selecting, altering and applying their other resources to meet stressful demands. Hence, those with higher level of self-efficacy could employ proactive coping behaviors more than those with lower efficacy beliefs could. Similarly, Jex et al. [27] suggested that individuals with high self-efficacy were more likely to believe they could maintain acceptable levels of performance despite the presence of job stressors and were more likely to use effective ways of coping with workplace stressors.

### 1.3. Proactive Coping

Proactive coping has often been related to stress [28]. “Proactive coping can be defined as an effort to build up general resources that facilitate promotion toward challenging goals and personal growth” ([29], p. 23). When facing distressful events, individuals who employ proactive coping strategies report lower stress [30]. Proactive coping involves anticipating future opportunities or problems, setting goals and planning how to achieve them, and implementing future-oriented plans in the face of obstacles, and can be treated as a personal resource [31]. It consists of efforts undertaken in advance of a potentially stressful event to prevent it or to modify its form and outcomes before it occurs [32]. Research has already documented that for the proactive individuals, coping is not a single response and can be conceptualized as a trait. It is an approach to life, a belief that things will work out not because of luck or other uncontrollable factors, but because the individual takes responsibility for outcomes [33]. In terms of COR theory, such behavior may prevent further loss of resources and can establish a gain cycle.

During school years, students diagnosed with learning disorders are entitled to and are often provided with learning and testing accommodations such as extended time. Yet when these individuals have to face the after-school competitive work environment, they often hesitate to inform others of their difficulties [34]. Some students with chronic academic challenges acquire active strategies that compensate their learning difficulties and enable them to demonstrate their abilities. They may develop proactivity in identifying ways to change the environmental conditions in order to meet their specific needs. Proactive coping is a personal resource that enables individuals to actively create desired environmental changes to compensate for personal needs and difficulties, and to demonstrate their abilities [35,36].

Proactive people do not only anticipate potentially harmful events, but also perceive them as opportunities to learn and grow. By positively reinterpreting stressful events as challenges rather than just threats, and in doing so fostering a positive motivation, they will be able to regulate their stress and face their difficulties [37]. Thus, it is not surprising that proactive coping predicts hopeful thinking regardless the severity of the individuals’ disability [38].

### 1.4. Hope Theory

Hope has been defined as the perceived ability to plan pathways to desired goals and motivate oneself via agentic thinking to advance those pathways [39]. In comparison to their lower-hope counterparts, individuals with higher hope identify ambitious yet attainable goals, consistently pursue them, and develop a greater number of effective paths for reaching them, 2018 [40]. Hope is conceptualized as two interrelated sub-constructs: (a) hope agency, and (b) hope pathways. Hope agency refers to individuals’ confidence in their ability to meet desired goals. Hope pathways involve the perception of available plans or strategies to reach the goals or to overcome barriers [39].

Considering the relations between the two sub-constructs, the current study has used the global measures of hope consisting of a combined agency and pathways measure. Based on these theoretical propositions and the evidence that hope is associated with successful goal outcomes, results suggest that higher levels of hope are associated with specific goal setting and goal-pursuit behaviors that may increase the likelihood of successful goal attainment and success among college students [41]. A recent longitudinal study revealed that adolescents’ hope predicted academic achievements [42]. Studies of students with academic difficulties at different age groups focused attention on the role of hope in predicting academic achievement and wellbeing [1,43,44].

### 1.5. The Present Study

Research demonstrated that students with prevalent learning difficulties (such as specific learning disorders or attention deficit disorders) often experience increased social distress and alienation from their peers in addition to their academic challenges during their school period. The entrance to higher education and/or work environments offers them new opportunities, while at the same time posing many new challenges. Some individuals have developed personal resources that can be relied on while confronting the new demands. Others have diluted their personal resources following their prolonged struggles.

Based on earlier studies, using COR theory [11], Bandura’s [45,46] social-cognitive theory, and hope theory [39], we predicted that higher self-efficacy will enhance proactive coping and higher levels of hope. While learning difficulties represent a distress, due to areas of deficit and difficulties, self-efficacy presents an option for change through a competence self-belief system, that has been developed through childhood and adults’ experiences. Bandura [24,46], in a comprehensive survey of studies, demonstrated that self-efficacy has to be considered as an operational power that produces processes while initiating and affecting desired changes. In our proposed model, based on recent generalized self-efficacy conceptualization [24,46,47], we predict that this self-belief will activate changes through two future oriented factors: a. the proactive coping that is focused on taking active steps to influence the environment in order to actualize preferred goals [36,48] and b. hope that is focused on the ability to plan pathways and motivate individuals towards achieving the desired goals—reduced loneliness [39]. Wang & Lei [36] study examined the sequence between proactivity and hope, and demonstrated in their research that proactive individuals tend to have higher hope (positive expectations for achieving goals, and to identify pathways toward achieving these goals). Similarly, we hypothesized that people who are more proactive coper, will develop higher levels of hope. These protective future oriented factors may mediate the distressful impact of learning difficulties on the loneliness and alienation.

Accordingly, we hypothesized as follows:

**Hypothesis** **1 (H1).** *Learning difficulties predicts loneliness.*

**Hypothesis** **2 (H2).** *Self-efficacy mediates the relations between learning difficulties and proactive coping.*

**Hypothesis** **3 (H3).** *Hope mediates the relations between learning difficulties, self-efficacy and proactive coping and levels of loneliness.*

## 2. Method

### Participants

The sample consisted of 498 Israeli participants (342 women—68.7%) at a mean age of 34.26 (*SD* = 8.86). 153 participants (30.7%) were students and 345 (69.3%) were graduates. The participants were divided into two age groups. The younger group consisted of 241 participants (182 women—75.5%) with an age range of 18–31 (*M* = 27.21, *SD* = 2.92). The older group consisted of 257 participants (160 women—62.3%) with an age range of 32–68 (*M* = 40.86, *SD* = 7.33). Age group comparisons revealed, as expected, more students in the younger group (102—66.7%) than in the older group (51—33.3%). Altogether, 275 participants (55.2%) reported that they were diagnosed with learning disorders: younger group—155 participants (56.4%) and older group—120 (43.6%); 243 were entitled to test accommodations (137—56.4% from the younger group and 106—43.6% from the older group). In order to define levels of learning difficulties that are relevant to school and work environment, we used 8 items from the Specific Learning Disorders Scale (SLDS) [49], and by applying cluster analysis, two groups were defined: 195 participants with high learning difficulties (*M* = 3.11, *SD* = 0.59) and 303 participants with low learning difficulties (*M* = 1.47, *SD* = 0.56). Only 10 participants declare that they did not have any learning difficulties.

## 3. Measures

### 3.1. Learning Difficulties

The Specific Learning Disorders Scale (SLDS) [49] is a 20-item measure developed for self-reporting SLDs based on the DSM-5 definition (American Psychiatric Association, 2013) [50], with items such as “I don’t have the patience to sit in one place”, and “It happens to me that I don’t remember what I read a short time ago”. Items are rated on a five-point Likert scale from 0 (*not at all*) to 4 (*very much*). For the current study, only eight items relevant for both school and work environments were selected. Cronbach’s alpha for the current sample was 0.83.

### 3.2. Self-Efficacy

The New General Self-Efficacy Scale (NGSE), an 8-item self-report measure, was used to measure general self-efficacy. It was developed in Hebrew and English and validated by Chen et al. [51]. Sample items include “I will be able to achieve most of the goals that I have set for myself”, and “Even when things are tough, I can perform quite well”. Items are rated on a five-point Likert scale from 1 (*strongly disagree*) to 4 (*strongly agree*). Cronbach’s alpha for the current sample was 0.88.

### 3.3. Proactive Coping Inventory

The Hebrew adaptation of the 14-item proactive coping inventory (PCI) [37,52] was used to assess proactive behavior. Items are rated on a five-point Likert scale from 1 (*not at all*) to 4 (*very much*), with items such as “I always try to find a way to work around obstacles; nothing really stops me”. Cronbach’s alpha for the current sample was 0.78.

### 3.4. Hope

The Hope Scale [39] assesses belief in one’s own ability to pursue desired goals and employ the strategies needed to achieve them, with items such as “I can think of many ways to achieve my goals in life”. The adaptation used in the current study [53] consists of six items to which individuals respond using a six-point Likert scale ranging from one (*none of the time*) to six (*all of the time*), where a higher score reflects a higher level of hope. In the current study, Cronbach’s alpha of 0.86 was obtained.

### 3.5. Loneliness

Loneliness was examined with the emotional and social loneliness scale [54] with items such as “I experience a general sense of emptiness” and “I miss having people around”. Responses for the 11-items scale were measured on a five-point Likert scale ranging from one (Very strongly disagree) to five (Very strongly agree). In the current study, a Cronbach’s alpha of 0.88 was obtained.

### 3.6. Procedure and Data Analysis

Prior to data collection, we obtained approval from the ethics committee of the college. (blinded for review). An invitation letter was sent to directors of two student support centers at Israeli colleges, asking for their help in disseminating the link to the questionnaire form to current and past students were entitled to test accommodations (using the Qualtrics system). Participation in this study was voluntary. Participants were not required to reveal personal details and were assured of anonymity. We adopted the terminology “learning difficulties” since the self-reported learning disorders and the symptoms of learning and attention disorders could not be validated by a clinical diagnosis due to privacy and anonymity issues.

The preliminary analysis consisted of an ANOVA to validate the short learning difficulties scale versus the self-reported SLDS; a three-ways MANOVA was used to examine the differences between participants with higher and lower learning difficulties who are students or graduates; and Pearson correlations were calculated to examine associations among the research measures. Based on models in which self-efficacy, proactive coping and hope were expected to mediate the relations between the levels of learning difficulties and loneliness, a mediation model was examined, using the Preacher and Hayes 4 [55] bootstrapping method with bias-corrected confidence estimates. In the present study, a 95% confidence interval for the indirect effects was obtained with 10,000 bootstrap resamples evaluating total, direct, and indirect effects. Age, gender, and education were kept as covariates [56]. Significance was determined by examining the 95% confidence interval produced by bootstrapping mediation analyses; this interval could not include zero or else the mediation model would be insignificant.

## 4. Results

### 4.1. Preliminary Analyses

Pearson correlations between the research measures are presented in Table 1. In order to validate the short scale of learning difficulties, we performed an ANOVA with the self-reported diagnosed SLD, being a student and gender as the independent variables, and the mean scores of learning difficulties as the dependent variable. The ANOVA yielded a main effect for the SLD group, *F* (1, 490) = 51.37, *p* < 0.01, partial *η*^2^ = 0.095; a main effect for being a student, *F* (1, 490) = 62.37, *p* < 0.01, partial *η*^2^ = 0.095; and an interaction between the SLD diagnosis and being a student *F* (1, 490) = 5.80, *p* < 0.01, partial *η*^2^ = 0.012 (students without SLD: *M* = 2.29, *SD* = 0.95; students with SLD: *M* = 2.91, *SD* = 0.74; not students without SLD: *M* = 1.37, *SD* = 0.71; not students with SLD: *M* = 2.37, *SD* = 0.83.

Means SDs and *F* scores are presented in Table 2.

The comparison between participants with SLD, showed that students reported more learning difficulties than not-students did, *F* (1, 271) = 29.12, *p* < 0.01, partial *η*^2^ = 0.097. The comparison between participants without SLD showed that students reported more learning difficulties than did not-students, *F* (1, 219) = 37.60, *p* < 0.01, partial *η*^2^ = 0.015. The comparison between students showed that participants with SLD reported more learning difficulties than the comparison group: *F* (1, 149) = 6.52, *p* < 0.01, partial *η*^2^ = 0.042. Finally, the comparison between participants who are graduates and not students anymore showed that participants with SLD reported more learning difficulties than the comparison group: *F* (1, 341) = 118.80, *p* < 0.01, partial *η*^2^ = 0.258).

In order to explore whether the two research groups (with high and low learning difficulties) differed in the research variables, we performed a three-way MANOVA, with learning difficulties × gender × student status serving as the independent variables, and loneliness, proactive coping, self-efficacy and hope as the dependent variables, and education level as a covariate. The MANOVA yielded a main effect for learning difficulties, *F* (4, 485) = 4.73, *p* < 0.01, partial *η*^2^ = 0.038; and a main effect for student status, *F* (4, 485) = 2.78, *p* < 0.05, partial *η*^2^ = 0.022. The gender and the remaining interactions were not significant. Means, SDs and *F* scores are presented in Table 3. The univariate analysis for learning difficulties yielded a main effect only for loneliness (low learning difficulties: *M* = 2.01, *SD* = 0.78; high learning difficulties: *M* = 2.36, *SD* = 0.84, *F* (1, 488) = 15.44, *p* < 0.01, partial *η*^2^ = 0.031). As expected, participants with higher levels of learning difficulties reported higher levels of loneliness, supporting H1. The remaining comparisons were not significant. The univariate analysis for the students’ status yielded a main effect for loneliness (students: *M* = 2.18, *SD* = 0.82; not students: *M* = 2.13, *SD* = 0.82, *F*(1, 488) = 4.68, *p* < 0.05, partial *η*^2^ = 0.01) and for proactive coping (students: *M* = 3.11, *SD* = 0.49; not students: *M* = 3.07, *SD* = 0.49, *F*(1, 488) = 6.56, *p* < 0.05, partial *η*^2^ = 0.01). Students reported higher levels of loneliness and proactive coping. The remaining comparisons were not significant.

In order to examine further the specific role of the predictors and their interrelations, a mediation analysis was performed to identify the mediating variables between the SLD groups and loneliness.

### 4.2. Mediation Analyses

In order to further examine whether the relations between levels of difficulty and loneliness could be accounted for by self-efficacy, proactive coping and hope, we performed a serial multiple mediation analysis, using model 6 in Hayes’ PROCESS (2018) [56] to identify indirect effects. The mediation analyses were tested using the bootstrapping method, with bias-corrected confidence estimates [55]. In the present study, a 95% confidence interval of the indirect effects was obtained with 10,000 bootstrap resamples [56].

**Figure 1:**
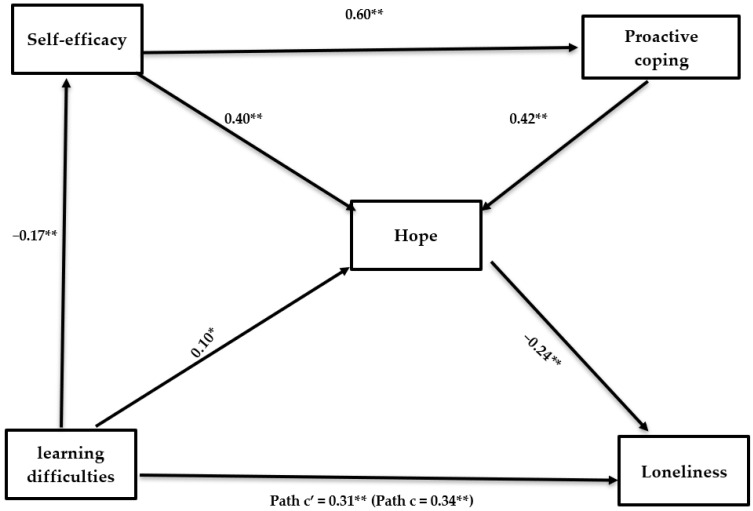
Direct and Indirect Effect of Learning Difficulties on Loneliness, Mediated by Self-Efficacy, Proactive Coping and Hope (Standardized Scores). Note. * *p* < 0.05; ** *p* < 0.01.

As presented in Table 4 and Figure 1, the total predictive effect of learning difficulties on loneliness was significant. Participants who had more learning difficulties also felt lonelier. In addition, self-efficacy, proactive coping, and hope mediated the relations between levels of difficulty and loneliness. As hypothesized, the relations between learning difficulties and proactive coping were mediated by self-efficacy. In addition, the relations between self-efficacy and proactive coping and loneliness were mediated by hope. The remaining paths were not significant.

Given that we entered the learning difficulties and the three mediating variables into the equation simultaneously, the relation between levels of difficulty and loneliness was reduced yet remained significant, thus supporting H2 and H3. In conclusion, the relation between experiencing learning difficulties and loneliness was explained not only directly (more learning difficulties predicted higher levels of loneliness), but also indirectly, through self-efficacy, proactive coping and hope as personal resources that reduced loneliness. The overall model was significant, *F* (10, 487) = 11.05, *p* < 0.001, explaining 18.5% of the total variance.

## 5. Discussion

The current study explored the loneliness experience of students and non-students with learning difficulties and the mediating role the personal resources of self-efficacy, proactive coping and hope in the relationship between learning difficulties and loneliness in college and beyond. In line with H1, the results demonstrated that young adults and adults with learning difficulties reported higher levels of loneliness compared with individuals without learning difficulties. Thus, the current study supports previous research documenting relations between younger students with learning disorders and loneliness among [20,21]. The current results demonstrated that adults continue to experience the academic distress during higher education and beyond.

Yet, the findings also suggested that individuals who were still students reported higher levels of learning difficulties compared to non-students. One explanation for this finding may be that during college learning difficulties are more pronounced and evident than after college. In order to succeed in their studies, they had to overcome attention difficulties and develop effective strategies to meet the demands of academic writing and testing [1]. Attention difficulties, slow learning, and memory challenges may also be manifested in life after college, albeit to a lesser degree [4].

Supporting H2 and H3, the results showed that self-efficacy, proactive coping and hope mediated the relations between levels of learning difficulty and loneliness. In line with COR theory [11], the social-cognitive theory [23] and hope theory [39], the current study demonstrated the importance of these personal resources in coping with loneliness both in college and beyond.

The identified model extended previous results that focused attention on the role of self-efficacy, proactive coping and hope as important coping resources both in academic settings (e.g., [1]) and beyond (e.g., [57,58]. Moreover, the positive relations between these resources demonstrated that the gain of resources established a virtuous cycle that created its own positive processes: individuals with higher self-efficacy benefited more in terms of both stronger proactive coping and higher hope. Special attention has to be devoted also to the meaning of coping for the proactive individuals. This should not be treated as a single response. It is an approach to life, an existential belief that things would work out not because of luck or other uncontrollable factors, but because the individual taking responsibility for outcomes [33]. Proactive people are able to create opportunities, show initiative, and persevere when facing obstacles, while reporting higher job satisfaction. Thus, in the current study, the mediating role of proactive coping between self-efficacy and hope emphasizes its importance [59].

Specifically, the final model emphasized the role of hopeful beliefs. Indeed, self-efficacy mediated the relations between learning difficulties and hope as well as between learning difficulties and proactive coping. Individuals with greater confidence in their competence tend to have both higher hope and stronger proactive coping. However, both self-efficacy and proactive coping, as well as learning difficulties, were mediated by hope, suggesting that having hopeful beliefs predicts reduced levels of loneliness. Lower levels of hope were predicted by the levels of difficulty, whereas higher levels of hope were predicted by levels of self-efficacy as an expression of self-competency and strength of proactive coping as an expression of dynamic and consistent energies to struggle with challenging situations. Higher levels of hope predicted the ability to identify goals and find paths to reach them. Thus, hopeful individuals are more active and consistent in investing efforts to overcome social distress and reduce their loneliness.

### 5.1. Theoretical Model and Implications

The proposed model demonstrates that only a comprehensive consideration of the interrelations among experiencing difficulties and different personal strengths may provide an in-depth understanding of the dynamic of loneliness. Such an integrated model has theoretical importance, presenting loneliness as a complex construct with multiple personal predictors. The final model emphasized the unique role of hopeful beliefs in constructing a meaningful life [60]. The findings of this study represent a contribution to the emerging field of positive psychology by demonstrating that beliefs in personal competence, as well as the ability to proactivity change situational challenges while promoting hopeful expectations and engagements are associated with psychological well-being, good health, meaning of life and reduced social stress [60,61,62].

A general theoretical model may be considered where individuals who have acquired greater confidence in their competence regardless their learning difficulties tend to have both higher hope and stronger proactive coping. However, both self-efficacy and proactive coping, as well as learning difficulties, were associate with hope, suggesting the need of hopeful beliefs in order to expect reduced loneliness. This model presents a dynamic construct, in which lower levels of hope were predicted by the levels of difficulty, whereas at the same time, higher levels of hope were predicted by levels of self-efficacy (as an expression of self-competency regardless the difficulties) and the strength of proactive coping (as an expression of dynamic and consistent engagement in struggling with challenging situations). The model integrates these constructs in a way that allows for the prediction of outcomes in various spheres of positive constructs, thus, providing a vehicle for future research in the area of positive psychology.

The results of the study have a clear psychological implication for research on transition of individuals to higher education and working environments, sensitizing attention to the risks of loneliness and high prevalence difficulties such as learning disorders and attention deficit disorders [63]. During school, many students with high prevalence disabilities are entitled to learning and testing accommodations. When they consider entering into the competitive working environment, they often worried if they will be able to adapt socially and professionally to the new demanding environments. The results of the study demonstrate that introducing future orientation personal strengths may reduce some of the social distress. Their self-efficacy, reflecting their beliefs in their competence, may serve as a source of inclination to proactively attempts to change some interfering aspects in their working and learning environments, and together may raise their hopeful expectations. As a result, this process may reduce the anxiety due to alienation expectations. This study is the first attempt to explore the role of proactivity in relations to hope and loneliness. It is a first step towards future longitudinal studies that will follow up the role enhancing self-efficacy, training proactivity and hope in securing a safer transition to work for individuals who experienced learning difficulties in school.

### 5.2. Limitations and Future Directions

Several limitations should be noted in interpreting the present findings. First, the study data were collected from a single source, possibly increasing the risk of common method variance [64]. Nonetheless, we used procedural design methods (confidentiality, anonymity, and separate instructions) to minimize this risk [65]. Future studies have to include in-depth interviews on personal experiences in order to explore different modes of processes between loneliness, proactivity and hopeful thinking.

In addition, the current study is cross-sectional and therefore it is not possible to determine causality in the described relationships between the variables. Based on the theoretical model we have examined the chosen configuration of the variables described in the present study, but other configurations and additional protective variables may be possible as well. Future studies should include longitudinal procedures to clarify processes leading to and maintaining loneliness.

A final limitation is the heterogeneous sample and the overrepresentation of women, and it was controlled in the analysis. Indeed, our goal was to include college students and graduate in order to explore their adaptation to different environments. Nonetheless, future research should include samples that are more balanced as well as longitudinal and experimental designs to examine the long-term predictive role of learning difficulties on loneliness while exploring the empowering roles of self-efficacy, proactivity and hope.

### 5.3. Intervention Implications

The results focused awareness on social and emotional distress that individuals with learning challenges may experience. Providing academic support and help to meet the academic needs is only a partial response. The findings support the need to teach and nurture hope in educational settings. Studies have already demonstrated that self-efficacy, proactive coping, and hopeful thinking are malleable and teachable constructs, and can be empowered and trained [33,66,67]. This is the first study that explore the role of proactivity as a mediator of loneliness and its relations with hope. Empowering proactive coping and hope can inspire students and graduates to set goals as well as provide them with a strategy of how to reach them through agency and pathway thinking. It is important to emphasize that college support centers, administrators, and staff all play a role in developing a community of hope in education systems. Special awareness is required for the affective processing of these individuals, their loneliness distress, hopeful beliefs, feelings of competency and proactive coping. To promote these personal resources, there is a need to sensitize professionals who provide support to students with learning difficulties in different age levels to the social and emotional needs of these students. In addition, Future intervention planning may consider embracing empowerment strategies in current academic support programs in order to enhance these strengths during higher education studies and in transition to working environments.

## 6. Conclusions

The study goals were to identify the role of self-efficacy, proactive coping, and hope as mediators in the relations between learning difficulties and loneliness distress. In line with the conservation of resources, social-cognitive and hope theories, we expected that individuals with learning difficulties will experience higher levels of loneliness compared with individuals without learning difficulties, focusing attention on mediating factors. The findings highlight the complexity of the loneliness construct by focusing attention on its relations not only with social distress, but also with sources of alienation due to academic difficulties. The current study focused attention on the emotional outcomes of individuals’ disturbing awareness of their chronic learning difficulties. The bitter experience of feeling different does not end with school years and may stimulate the alienation experience, let alone when accompanied by peer rejection. However, the results also demonstrated the importance of the protective role played by self-efficacy, proactive coping and hope in mediating the relations between learning difficulties and social distress. Professionals’ sensitizing to their value, as well as interventions and psychoeducational programs should be tailor-made to enhance more hopeful beliefs and reduced loneliness.

## Figures and Tables

**Table 1:** Pearson Correlations of the Research Variables.

*N* = 498	1	2	3	4	5	6	7
1. Age	________						
2. Learning difficulties	−0.12 **	______					
3. Education	0.23 **	−0.21 **	_______				
4. Loneliness	0.01	0.23 **	−0.12 *	________			
5. Self-efficacy	0.12 *	−0.16 **	0.09 *	−0.28 **	________		
6. Proactive coping	0.16 **	−0.07	0.09	−0.27 **	0.60 **	________	
7. Hope	0.07	0.01	0.06	−0.32 **	0.63 **	0.64 **	_______
*M*	34.26	2.12	1.91	2.15	3.21	3.08	4.45
*SD*	0.86	0.98	0.98	0.82	0.43	0.49	0.78

Note. * *p* < 0.05; ** *p* < 0.01.

**Table 2:** Means, SD and *F* scores of SLD and being a student as independent variables and learning difficulties as the dependent variable.

		M (SD)	Comparisons
Not SLD ^1^	a. Student	2.29 (0.95)	a > b
	b. Not student	1.37 (0.71)	
SLD ^1^	c. Student	2.91 (0.74)	c > d
	d. Not Student	2.37 (0.83)	

Notes: ^1^ SLD—Specific Learning Disorders; Main effect for SLD: *F* (1, 490) = 51.37, *p* < 0.01, partial *η*^2^ = 0.095, SLD reported more difficulties. Main effect for students: *F* (1, 490) = 62.37, *p* < 0.01, partial *η*^2^ = 0.095, Students reported more difficulties. Interaction SLD X students: *F* (1, 490) = 5.80, *p* < 0.01, partial *η*^2^ = 0.012, c, d > a, b; c > a, d > b.

**Table 3:** Means and SDs of the dependent variables: Loneliness, Proactive coping, Self-efficacy and Hope among groups of learning difficulties and students’ status.

	Learning Difficulties		F (1, 488)	Students		F (1, 488)
	Low (N = 303) M (SD)	High (N = 195) M (SD)		Yes (N = 153)	No (N = 345)	
Loneliness	2.01 (0.78)	2.36 (0.81)	23.74 ** (0.046)	2.18 (0.82)	2.13 (0.82)	4.68 * (0.01)
Proactive coping	3.11 (0.55)	3.03 (0.47)	2.69 (0.01)	3.11(0.49)	3.07 (0.49)	6.56 * (0.01)
Self-efficacy	3.25 (0.43)	3.15 (0.43)	2.42 (0.01)	3.17 (0.42)	3.23 (0.45)	0.01 (0.00)
Hope	4.46 (0.82)	4.44(0.72)	0.03 (0.01)	4.47 (0.72)	4.44 (0.81)	1.21 (0.00)

Notes: * *p* < 0.05, ** *p* < 0.01.

**Table 4:** Direct and Indirect Effects of Learning Difficulties on Loneliness.

Direct Path	Standardized Coefficient	SE	*t*	LLCI	ULCI
Learning difficulties to Loneliness (before mediation)—Path c	0.34 **	0.05	5.95 **	0.1869	0.3711
Learning difficulties to Self-efficacy	−0.17 **	0.03	−2.93 **	−0.1234	−0.0242
Self-efficacy to Proactive coping	0.60 **	0.04	16.55 **	0.5955	0.7560
Self-efficacy to Hope	0.40 **	0.07	9.98 **	0.5789	0.8628
Proactive coping to Hope	0.42 **	0.06	10.39 **	0.5392	0.7908
Learning difficulties to Hope	0.10 *	0.03	2.28 *	0.0104	0.1387
Hope to Loneliness	−0.24 **	0.06	−3.99 **	−0.3690	−0.1254
Learning difficulties to Loneliness (after mediation)—Path c’	0.31 **	0.05	5.72 **	0.1689	0.3458

Note. LLCI—Lower limit confidence interval; ULCI—Upper limit confidence interval. * *p* < 0.05; ** *p* < 0.01.

## Data Availability

The data can be obtained by mail from the corresponding author malka@tauex.tau.ac.il.

## References

[1] Mana A., Saka N., Dahan O., Ben-Simon A., Margalit M. (2021). Implicit theories, social support, and hope as serial mediators for predicting academic self-efficacy among higher education students. Learn. Disabil. Q..

[2] Russak S., Hellwing D.A. (2015). A follow-up study of graduates with learning disabilities from a college of education: Impact of the disability on personal and professional life. Aust. J. Learn. Difficult..

[3] Klassen R.M., Tze V.M., Hannok W. (2013). Internalizing problems of adults with learning disabilities: A meta-analysis. J. Learn. Disabil..

[4] Gerber P.J. (2012). The impact of learning disabilities on adulthood: A review of the evidenced-based literature for research and practice in adult education. J. Learn. Disabil..

[5] Rokach A., Berman D., Rose A. (2021). Loneliness of the blind and the visually impaired. Front. Psychol..

[6] Margalit M. (2012). Lonely Children and Adolescents: Self Perceptions, Social Exclusion and Hope.

[7] Einav M., Sharabi A., Even-hen Peter T., Margalit M. (2018). Test accommodations and positive affect among adolescents with learning disabilities: The mediating role of attitudes, academic self-efficacy, loneliness and hope. Athens J. Educ..

[8] Heiman T., Olenik Shemesh D. (2020). Social-emotional profile of children with and without learning disabilities: The relationships with perceived loneliness, self-efficacy and well-being. Int. J. Environ. Res. Public Health.

[9] Emerson E., Fortune N., Llewellyn G., Stancliffe R. (2021). Loneliness, social support, social isolation and wellbeing among working age adults with and without disability: Cross-sectional study. Disabil. Health J..

[10] Pressman S.D., Cohen S., Miller G.E., Barkin A., Rabin B.S., Treanor J.J. (2005). Loneliness, social network size, and immune response to influenza vaccination in college freshmen. Health Psychol..

[11] Hobfoll S.E. (2002). Social and psychological resources and adaptation. Rev. Gen. Psychol..

[12] Buecker S., Maes M., Denissen J.J.A., Luhmann M. (2020). Loneliness and the Big Five Personality Traits: A meta–analysis. Eur. J. Personal..

[13] Hobfoll S.E., Halbesleben J., Neveu J.-P., Westman M. (2018). Conservation of resources in the organizational context: The reality of resources and their consequences. Annu. Rev. Organ. Psychol. Organ. Behav..

[14] Nielsen K., Nielsen M.B., Ogbonnaya C., Känsälä M., Saari E., Isaksson K. (2017). Workplace resources to improve both employee well-being and performance: A systematic review and meta-analysis. Work. Stress.

[15] Peplau L.A., Perlman D. (1982). Loneliness: A Sourcebook of Current Theory, Research, and Therapy.

[16] von Soest T., Luhmann M., Hansen T., Gerstorf D. (2020). Development of loneliness in midlife and old age: Its nature and correlates. J. Personal. Soc. Psychol..

[17] Barreto M., Victor C., Hammond C., Eccles A., Richins M.T., Qualter P. (2021). Loneliness around the world: Age, gender, and cultural differences in loneliness. Personal. Individ. Differ..

[18] Holt-Lunstad J. (2021). The major health implications of social connection. Curr. Dir. Psychol. Sci..

[19] Cigna (2020). Loneliness and Its Impact on the American Workplace: Understanding the Drivers of Workplace Loneliness, the Costs and the Solutions. https://www.cigna.com/static/www-cigna-com/docs/about-us/newsroom/studies-and-reports/combatting-loneliness/loneliness-and-its-impact-on-the-american-workplace.pdf.

[20] Majorano M., Brondino M., Morelli M., Maes M. (2017). Quality of relationship with parents and emotional autonomy as predictors of self-concept and loneliness in adolescents with learning disabilities: The moderating role of the relationship with teachers. J. Child Fam. Stud..

[21] Musetti A., Eboli G., Cavallini F., Corsano P. (2019). Social relationships, self-esteem, and loneliness in adolescents with learning disabilities. Clin. Neuropsychiatry.

[22] Qualter P., Vanhalst J., Harris R., Van Roekel E., Lodder G., Bangee M., Maes M., Verhagen M. (2015). Loneliness across the life span. Perspect. Psychol. Sci..

[23] Bandura A. (1997). Self-Efficacy: The Exercise of Control.

[24] Bandura A. (2018). Toward a psychology of human agency: Pathways and reflections. Perspect. Psychol. Sci..

[25] Stajkovic A.D., Bandura A., Locke E.A., Lee D., Sergent K. (2018). Test of three conceptual models of influence of the big five personality traits and self-efficacy on academic performance: A meta-analytic path-analysis. Personal. Individ. Differ..

[26] Al-Yagon M., Margalit M., Levesque J.R.R. (2016). Specific learning disorder. Encyclopedia of Adolescence.

[27] Jex S.M., Bliese P.D., Buzzell S., Primeau J. (2001). The impact of self-efficacy on stressor-strain relations: Coping style as an explanatory mechanism. J. Appl. Psychol..

[28] Schwarzer R. (2001). Stress, resources, and proactive coping. Appl. Psychol. Int. Rev..

[29] Schwarzer R., Luszczynska A. (2008). Reactive, anticipatory, preventive, and proactive coping: A theoretical distinction. Prev. Res..

[30] Gillespie G.L., Gates D.M. (2013). Using proactive coping to manage the stress of trauma patient care. J. Trauma Nurs..

[31] Searle B.J., Lee L. (2015). Proactive coping as a personal resource in the expanded job demands–resources model. Int. J. Stress Manag..

[32] Bui T.H.T., Nguyen T.N.T., Pham H.D., Tran C.T., Ha T.H. (2021). The mediating role of self-compassion between proactive coping and perceived stress among students. Sci. Prog..

[33] Parada S., Verlhiac J.-F. (2021). Growth mindset intervention among French university students, and its articulation with proactive coping strategies. Educat. Psychol..

[34] Spirito Dalgin R., Bellini J. (2008). Invisible disability disclosure in an employment interview: Impact on employers’ hiring decisions and views of employability. Rehabil. Couns. Bull..

[35] Bateman T.S., Crant J.M. (1993). The proactive component of organizational behavior: A measure and correlates. J. Organ. Behav..

[36] Wang H., Lei L. (2021). Proactive personality and job satisfaction: Social support and hope as mediators. Curr. Psyhol..

[37] Greenglass E.R., Frydenberg E. (2002). Proactive coping and quality of life management. Beyond coping: Meeting Goals, Visions, and Challenges.

[38] Phillips B.N., Smedema S.M., Fleming A.R., Sung C., Allen M.G. (2016). Mediators of disability and hope for people with spinal cord injury. Disabil. Rehabil..

[39] Snyder C.R. (2002). Hope theory: Rainbows in the mind. Psychol. Inq..

[40] Gallagher M.W., Lopez S.J. (2018). The Oxford Handbook of Hope.

[41] Cheavens J.S., Heiy J.E., Feldman D.B., Benitez C., Rand K.L. (2019). Hope, goals, and pathways: Further validating the hope scale with observer ratings. J. Posit. Psychol..

[42] Fraser A.M., Bryce C.I., Alexander B.L., Fabes R.A. (2021). Hope levels across adolescence and the transition to high school: Associations with school stress and achievement. J. Adolesc..

[43] Laslo-Roth R., Bareket-Bojmel L., Margalit M. (2021). Loneliness experience during distance learning among college students with ADHD: The mediating role of perceived support and hope. Eur. J. Spec. Needs Educ..

[44] Sainio P.J., Eklund K.M., Ahonen T.P.S., Kiuru N.H. (2019). The role of learning difficulties in adolescents’ academic emotions and academic achievement. J. Learn. Disabil..

[45] Bandura A. (1977). Social Learning Theory.

[46] Bandura A. (2019). Applying theory for human betterment. Perspect. Psychol. Sci..

[47] Tam L., Choi S.-h., Kim J.-N. (2021). Reconceptualizing the self-efficacy construct in public relations research: The case of sojourners and their communicative behaviors. Public Relat. Rev..

[48] Schwarzer R., Taubert S., Frydenberg E. (2002). Tenacious goal pursuits and striving toward personal growth: Proactive coping. Beyond Coping: Meeting Goals, Visions and Challenges.

[49] Rosenblat R., Margalit M. (2018). Specific Learning Disorders Scale.

[50] American Psychiatric Association (2013). DSM-5: Diagnostic and Statistical Manual of Mental Disorders, 5th ed.

[51] Chen G., Gully S.M., Eden D. (2001). Validation of a new general self-efficacy scale. Organ. Res. Methods.

[52] Etzion D., Greenglass E. (2004). The Proactive Coping Inventory (PCI): Hebrew Version. http://userpage.fu-berlin.de/~health/pci_hebrew.htm.

[53] Lackaye T., Margalit M. (2006). Comparisons of achievement, effort and self-perceptions among students with learning disabilities and their peers from different achievement groups. J. Learn. Disabil..

[54] De Jong-Gierveld J., Van Tilburg T.G. (2006). A 6-Item Scale for overall, emotional, and social loneliness: Confirmatory tests on survey data. Res. Aging.

[55] Preacher K., Hayes A. (2004). SPSS and SAS procedures for estimating indirect effects in simple mediation models. Behav. Res. Methods Instrum. Comput..

[56] Hayes A.F. (2018). Introduction to Mediation, Moderation and Conditional Process Analysis.

[57] Cruz J.P., Cabrera D.N.C., Hufana O.D., Alquwez N., Almazan J. (2018). Optimism, proactive coping and quality of life among nurses: A cross-sectional study. J. Clin. Nurs..

[58] Vagni M., Maiorano T., Giostra V., Pajardi D. (2020). Coping with COVID-19: Emergency stress, secondary trauma and self-efficacy in healthcare and emergency workers in Italy. Front. Psychol..

[59] Stanojević D., Krstić M., Jaredić B., Dimitrijević B. (2014). Proactive coping as a mediator between resources and outcomes: A structural equations modeling analysis. Appl. Res. Qual. Life.

[60] Feldman D., Balaraman M., Anderson C., Gallagher M.G., Lopez S.J. (2018). Hope and Meaning-in-Life. Points of Contact Between Hope Theory and Existentialism. The Oxford Handbook of Hope.

[61] Feldman D.B., Snyder C.R. (2005). Hope and the meaningful life: Theoretical and empirical associations between goal-directed thinking and life meaning. J. Soc. Clin. Psychol..

[62] Greenglass E.R., Fiksenbaum L. (2009). Proactive coping, positive affect, and well-being: Testing for mediation using path analysis. Eur. Psychol..

[63] Hakkarainen A.M., Holopainen L.K., Savolainen H.K. (2016). The impact of learning difficulties and socioemotional and behavioural problems on transition to postsecondary education or work life in Finland: A five-year follow-up study. Eur. J. Spec. Needs Educ..

[64] Spector P.E. (2006). Method variance in organizational research: Truth or urban legend?. Organ. Res. Methods.

[65] Podsakoff P.M., MacKenzie S.B., Lee J.-Y., Podsakoff N.P. (2003). Common method biases in behavioral research: A critical review of the literature and recommended remedies. J. Appl. Psychol..

[66] Bartimote-Aufflick K., Bridgeman A., Walker R., Sharma M., Smith L. (2016). The study, evaluation, and improvement of university student self-efficacy. Stud. High. Educ..

[67] Feldman D.B., Davidson O.B., Margalit M. (2015). Personal resources, hope, and achievement among college students: The conservation of resources perspective. J. Happiness Stud..

